# Immune profiles of elderly breast cancer patients are altered by chemotherapy and relate to clinical frailty

**DOI:** 10.1186/s13058-017-0813-x

**Published:** 2017-02-28

**Authors:** Jithendra Kini Bailur, Graham Pawelec, Sigrid Hatse, Barbara Brouwers, Ann Smeets, Patrick Neven, Annouschka Laenen, Hans Wildiers, Christopher Shipp

**Affiliations:** 1 Department of Internal Medicine II, University Hospital Tübingen, Waldhörnlestr. 22, 72072 Tübingen, Germany; 2 Present: Yale Cancer Center, Yale University School of Medicine, New Haven, 06510 CT USA; 3 Laboratory of Experimental Oncology (LEO), Department of Oncology, KU Leuven, Leuven, Belgium; 4 Department of General Medical Oncology, University Hospital Leuven, Leuven Cancer Institute, Leuven, Belgium; 5 Leuven Multidisciplinary Breast Center, University Hospital Leuven, Leuven, Belgium; 6 Interuniversity Center for Biostatistics and Statistical Bioinformatics, Leuven, Belgium

**Keywords:** Breast cancer, Immune profile, Chemotherapy, Clinical frailty, Blood leukocyte

## Abstract

**Background:**

Effective therapeutic management of elderly patients with cancer, on an individual basis, remains a clinical challenge. Here, we identify novel biomarkers to assess elderly patients (≥70 years of age) with breast cancer undergoing treatment with or without chemotherapy.

**Methods:**

We performed comprehensive geriatric assessment and measured markers sensitive to alteration in ageing, including leukocyte telomere length, CMV serostatus, levels of circulating growth factors and cytokines, and immune profiling of T cell and myeloid populations in blood before and at 3 months and 12 months after initiation of therapy, using flow cytometry.

**Results:**

We observed changes in immune profiles over time that were specific to patients receiving chemotherapy; these patients had elevated CD4+ T effector memory re-expressing CD45RA (TEMRA) cells and relatively lower CD8+ central memory cells at 3 months, with normalized levels after 12 months. Patients’ baseline immune profiles correlated with markers such as telomere length, cytomegalovirus (CMV) serostatus and levels of circulating cytokines. We also identified correlations between baseline immune profile and geriatric assessment, i.e. more frail patients had higher levels of granulocytic cells but lower levels of cells with suppressor phenotypes including myeloid-derived suppressor cells and regulatory T cells, although none of the examined immune populations correlated with chronological age. Importantly, immune profiles prior to therapy predicted unexpected hospitalizations in patients receiving chemotherapy.

**Conclusion:**

These findings suggest that immune profiling may represent a novel complementary approach to more accurately assess the global health status of the elderly patient with breast cancer and select the most appropriate individual treatment option.

**Trial registration:**

ClinicalTrials.gov, NCT00849758. Registered on 20 February 2009.

**Electronic supplementary material:**

The online version of this article (doi:10.1186/s13058-017-0813-x) contains supplementary material, which is available to authorized users.

## Background

Breast cancer is one of the most common cancers in elderly women, but only recently have steps been taken to standardize treatments for older patients [1, 2]. The high variability of individual health status in the elderly constitutes a major challenge in offering optimal therapy to this population. A comprehensive geriatric assessment (GA) allows an evaluation of the general health status of older patients, and predicts treatment toxicity and outcome to some extent [3], but it is still an imperfect, time-consuming tool and there is a lack of consensus on which specific geriatric scales should be used. Based on our own findings, biological markers of ageing and frailty could provide added value to clinical geriatric evaluation for assessing individual patients’ fitness [4].

Age-associated immune dysfunction or “immunosenescence” decreases the ability of older people to mount immune responses and results in inappropriate and prolonged inflammatory reactions, which may contribute to the development and/or progression of cancer. Hallmarks of an aged immune system are increased output of myeloid cells from the bone marrow and a concomitant decrease in B cells and T cell progenitors. Predominantly because of thymic involution early in life and exposure to pathogens throughout life (especially persistent viruses such as cytomegalovirus (CMV)), the older person’s immune system comprises quantitatively different cell types relative to younger people, with a predominance of memory T cells and deficit of naïve cells that have not yet been exposed to their cognate antigen. Moreover, immune cells may be functionally compromised in older people, with some showing signs of cellular senescence (short telomeres, accumulated DNA damage, and increased expression of cell-type-restricted surface molecules such as CD57 on CD8+ T cells).

Although not specific, the surface marker constellations measured on the T cell surface by flow cytometry can provide information about the “age” of that individual’s immune system [5]. Exposure to chemotherapy may exacerbate this immune ageing effect in elderly patients with cancer and render them more susceptible to diseases associated with dysregulated immunity [6]. Depending on the kinetics of the response, chemotherapeutic agents may also influence T cell-mediated anti-cancer responses, which can be assessed as functional “immune signatures” [7, 8]. However, the impact of chemotherapy on immunosenescence has been poorly investigated. Here, we investigated markers of immunosenescence in an older population of patients with breast cancer, who were receiving adjuvant chemotherapy, and compare these changes to patients with breast cancer who were not receiving chemotherapy, in order to examine chemotherapy-dependent effects on the immune system. Additionally, we tested correlation between these immune changes and clinical frailty defined by geriatric assessment, and other potential ageing biomarkers such as telomere length in white blood cells and circulating inflammatory cytokines, and assess the capacity of immune biomarkers to predict patient outcome following chemotherapy.

## Methods

### Patient population

This is a biomarker sub-study from a previously reported prospective clinical study evaluating the effect of adjuvant chemotherapy on clinical and biological ageing parameters in older female patients (≥70 years) with breast cancer [9] (trial registration number NCT00849758 (www.clinicaltrials.gov)). In brief, this was a prospective, multicenter, non-interventional study in which patients were recruited at two academic and two regional hospitals in Belgium between 2009 and 2012. Eligible patients were women aged 70 years or older with early invasive breast cancer, who were to receive adjuvant chemotherapy (4 × docetaxel-cyclophosphamide (TC)) (chemotherapy group (CTG)). It should be pointed out that TC was combined with trastuzumab in human epidermal growth factor receptor 2 (HER2)-positive patients in the CTG. All therapy was administered according to established risk factors and international guidelines [2]. In parallel, a control group consisted of patients with early breast cancer (≥70 years) in whom adjuvant chemotherapy was not indicated, and who were treated with an aromatase inhibitor as the sole adjuvant systemic therapy (control group (CG)). Written informed consent was obtained from all patients and the study was approved by the ethics committees of the participating hospitals.

Patients were enrolled after surgery and underwent blood sampling and full geriatric assessment (GA) and quality of life (QoL) evaluation at baseline, after 3 months and after 1 year. The first time point for blood draw was typically between 3 and 6 weeks after surgery, and always before administration of the first TC cycle in the CTG. The second time point was on the day of the fourth and last TC cycle (blood was taken immediately prior to chemotherapy administration) for patients in the CTG and approximately 3 months after inclusion for patients in the CG. The last time point was roughly 1 year after inclusion for both groups, or on the day before eligible patients received the 18th and last dose of trastuzumab.

GA was performed on all patients by a trained nurse. We performed a G8 screening test in all patients at baseline, and a full GA in all patients at the three time points as previously described [10]. In brief, the full GA consisted of social data, functional status assessed by Katz’s activities of daily living (ADL) and by Lawton’s instrumental activities of daily living (IADL) scales, fall history, self-perceived fatigue assessed by the mobility–tiredness test (MOB-T), cognitive status assessed by the mini mental state examination (MMSE), mood assessed by the 15-item geriatric depression scale (GDS-15), nutritional status assessed by the mini nutritional assessment-short form (MNA-SF) and comorbidity assessed by the Charlson comorbidity index (CCI).

Classical oncological parameters such as Eastern Cooperative Oncology Group-performance status (ECOG-PS) [10], tumor characteristics (tumor molecular subtype according to the St. Gallen criteria, and tumor, node and metastasis (TNM) staging) and treatment details and toxicity were also recorded. Pain was evaluated in every patient using a visual analog scale (VAS). Polypharmacy was assessed by the number of different registered drugs (http://www.bcfi.be/nl/start) the patient was using during the week preceding study inclusion. Quality of life was assessed with the European Organization for Research and Treatment for Cancer quality of life questionnaire (EORTC QLQ-C30) at the three time points. Combined GA results were categorized according to the Leuven oncology frailty score (LOFS), which summarizes GA results in a single score, ranging from 10 (very fit) to 0 (very frail) [9]. Overall clinical health was similar in the CG and CTG groups, although the controls had slightly worse clinical health profiles. Additional file 1 provides a detailed description of these cohorts.

### Immunophenotyping of blood leukocyte populations

The initial biomarker study [9] comprised 57 patients in the CTG and 52 controls. Frozen blood samples of sufficient quality for immune phenotyping were available at the three defined time points in 28 patients in each group. Collected whole blood was stored at −80 °C as previously described [11]. Briefly, dimethyl sulfoxide (DMSO) was added to each tube in a drop-wise manner to a final concentration of 10%; the solution was mixed well by gentle agitation and transferred to a plastic 15 ml tube. Samples were immediately placed at −80 °C before being shipped to Tübingen for storage in liquid nitrogen until use.

To perform immunophenotyping, frozen samples were thawed in a 37 °C water bath and immediately washed with a 24-fold excess of cold PBS supplemented with 2.0 mM EDTA (Serva, Heidelberg, Germany) and 2% fetal bovine serum (FBS). The diluted blood was gently inverted by hand three to four times to mix before undergoing centrifugation (×300 g at room temperature for 5 minutes). The supernatant was aspirated by pipette and samples were then treated with lysis buffer (BD Biosciences, Heidelberg, Germany) for 15 minutes and washed with phosphate buffered saline, 2% FBS, 2 mM EDTA and 0.01% azide (PFEA).

Before staining for different immune populations, Fc receptors were blocked with Gamunex (Bayer, Leverkusen, Germany) and dead cells labelled with the DNA-binding dye Ethidium Monoazide Bromide (Biotium, Hayward, USA) at 4 °C under bright light for 20 minutes in a single step. Then, 1.5 ml of diluted blood was used for each panel of fluorescently-labeled antibodies, combining markers of different cell types as follows: for T-cells: CD3-Alexa Fluor700, CD4-PerCp, CD8-APC-H7, CCR7-PO, CD27-APC, CD28-PE, CD45RA-V450, CD57-FITC and CD95PE-Cy7; for regulatory T cells (Tregs): surface CD4-PerCp, CD8-APC-H7, CD45RA-BV421, CD25-PE followed by intracellular staining of CD3-PO, FOXP3-Alexa-647 and Helios-FITC; for myeloid-derived suppressor cells (MDSCs): lineage cocktail (Lin) markers (CD3, CD19, CD56)-BV-605 along with the MDSC markers CD14-BV711, CD15-FITC, CD11b-APC-Cy7, CD33-Alexa-Fluor700, CD45-V500, CD124-PE and HLA-DR-PerCp-Cy5.5. Additional files 2, 3 and 4 show the gating strategy used and Additional file 5 displays a full list of leukocyte populations examined along with a non-technical explanation of the major marker proteins used.

### Measurement of CMV serostatus, plasma cytokines and chemokines and leukocyte telomere lengths

Blood was collected at 3 time points in 4 ml EDTA K2E tubes for plasma isolation and leukocyte DNA extraction. Blood tubes were centrifuged within 1 hour at × 1300 g at 4 °C for 10 minutes, and plasma was aliquoted for storage at –80 °C. The buffy coat of the plasma tube was kept at –20 °C for a maximum of 4 months prior to DNA extraction for telomere length assessment.

The following biomarkers were assessed as previously described [9]: mean leukocyte telomere length (TL), CMV serostatus, circulating inflammatory cytokines and growth factors IL-6, IL-10, insulin-like growth factor (IGF)-1, TNF-alpha, monocyte chemoattractant protein (MCP)-1 and regulated on activation, normal T cell expressed and secreted (RANTES).

### Endpoints

The primary aim of this biomarker study was to investigate how immune cell biomarkers (T cells, Tregs and myeloid-derived suppressor cells (MDSCs)) evolve over time in elderly patients with breast cancer on chemotherapy and whether this is different from control patients. Second, in all patients we assessed correlation between immune biomarkers (T cells, Tregs, MDSCs) with CMV status at inclusion (because whether or not the patient is infected with CMV has a major impact on the distribution of immune cells in the blood), age at diagnosis, clinical ageing status (G8 screening tool and a summarized scale of a full GA, the LOFS), telomere length and circulating inflammatory and anti-inflammatory cytokines (IL-6, IL-10, IGF-1, TNF, MCP-1 and RANTES). Third, in the chemotherapy group we also assessed whether immune biomarkers measured at inclusion predict decline in functionality (decline by 1 point or more in IADL at 1 year) and quality of life (10 points or more decrease at 1 year) in elderly patients receiving chemotherapy. Finally, we also investigated whether immune biomarkers measured at inclusion predict chemotherapy-induced grade II-III-IV toxicity and/or unexpected hospitalizations during the 3-month treatment period.

### Statistics

Continuous immune biomarkers were modeled by linear models and CMV by a logistic regression model, with time, study arm and their interaction as explanatory variables. An unstructured residual covariance matrix was modeled to account for clustering by repeated measures. Outcomes were transformed where needed, to improve the symmetry of distribution of the residuals. Spearman correlation was tested to study the association between immune biomarkers and continuous or ordinal variables. The Kruskal-Wallis test was used to compare biomarkers among more than two groups. The Mann-Whitney *U* test was used to compare two groups. Fisher’s exact test was used to study the association between CMV and categorical variables. All tests were two-sided with a significance threshold of 5%. This was a hypothesis-generating study that did not necessitate correction for multiple testing. As such, all *p* values are exploratory and require validation in an independent cohort.

## Results

### Impact of chemotherapy on the immune profile of elderly patients with breast cancer

To investigate therapy-dependent effects on the immune system, we monitored the immune profiles of elderly patients with breast cancer in the chemotherapy group (CTG) and the control group (CG) (28 patients in each group). Peripheral immune profiles were measured prior to receiving treatment and 3 months and 12 months after starting therapy. Patients receiving chemotherapy had increased frequencies of total CD8+ T cells (mostly cytotoxic T cells) after 3 months, resulting in a decreased ratio of CD4:CD8 (Table 1). In the analysis of suppressor leukocytes, two major populations of MDSCs (CD14 + HLA-DR– and CD14 + CD124+), but not Tregs, were increased at 3 months. In contrast to these observations at 3 months, naïve CD8+ T cells were elevated in patients in the CTG group after 12 months, while the two MDSC populations that had increased by 3 months remained elevated at 12 months. Furthermore, the elevated CD4:8 ratio observed at 3 months was normalized after 12 months. Table 1 provides a full list of leukocyte populations that were altered in patients receiving chemotherapy. Graphical results for these populations can be found in Additional file 6. Background information on these leukocyte populations can be found in Additional file 5.

**Table 1:** Changes in immune profile in patients undergoing chemotherapy

	Inclusion vs 3 months (CTG)		Inclusion vs 1 year (CTG)		Time by group interaction
	Mean difference (95% CI)	*p* Value	Mean difference (95% CI)	*p* Value	*p* Value
CD4 + CD27+	–1.64 (–7.27; 3.98)	0.561	8.34 (1.53; 15.15)	**0.017**	0.317
CD4 + CD28+	–2.11 (–6.33; 2.11)	0.321	5.78 (0.71; 10.85)	**0.026**	**0.018**
CD4 + CD27 + CD28+	–0.55 (–5.92; 4.81)	0.836	8.72 (2.22; 15.22)	**0.009**	0.328
CD4+/central memory	2.65 (–0.57; 5.88)	0.105	6.44 (3.4; 9.48)	**<0.001**	0.114
CD4 + CD27–	0.34 (0.11; 0.57)	**0.005**	–0.26 (–0.47; –0.04)	**0.020**	**<0.001**
CD4+/TEMRA	0.23 (0.03; 0.43)	**0.028**	–0.09 (–0.28; 0.1)	0.343	**0.008**
CD8+	7.69 (2.62; 12.75)	**0.004**	4.04 (–0.72; 8.8)	0.095	0.116
CD8 + CD27+	–4.77 (–8.8; –0.74)	**0.021**	2.10 (–1.85; 6.06)	0.292	**0.008**
CD8 + CD28+	–5.53 (–11.3; 0.24)	0.060	3.99 (–2.84; 10.83)	0.246	**0.011**
CD8 + CD27 + CD28+	–4.31 (–8.15; –0.46)	**0.029**	3.91 (0.62; 7.2)	**0.021**	**0.004**
CD8 + CD27-CD28–	6.35 (1.78; 10.92)	**0.007**	–4.42 (–9.18; 0.35)	0.068	**<0.001**
CD8+/central memory	–0.15 (–0.35; 0.05)	0.130	0.27 (0.07; 0.48)	**0.009**	**0.01**
CD8+/naive	–0.16 (–0.40; 0.09)	0.204	0.29 (0.06; 0.52)	**0.014**	0.151
CD4/CD8 ratio	–0.19 (–0.32; –0.07)	**0.003**	0.02 (–011; 0.15)	0.757	**0.022**
CD4 + CD25hi FOXP3+	–0.02 (–0.19; 0.15)	0.857	0.17 (0.02; 0.33)	**0.028**	0.185
CD4 + Helios+	–0.07 (–1.14; 1.01)	0.903	0.99 (0.18; 1.8)	**0.017**	0.511
Lin-CD14+	0.20 (–0.07; 0.47)	0.151	0.58 (0.33; 0.83)	**<0.001**	**0.021**
CD14 + HLA-DR–	0.13 (0.01; 0.26)	**0.039**	0.21 (0.08; 0.34)	**0.002**	0.591
CD14 + CD124+	0.06 (0.01; 0.11)	**0.014**	0.07 (0.02; 0.12)	**0.009**	0.092
CD15+	0.44 (0.04; 0.83)	**0.031**	–0.57 (–1.15; 0.01)	0.054	**0.018**

Mean differences are shown in measurements made at inclusion and at 3 months (n = 28) and at inclusion and at 1 year (n = 28) in the chemotherapy group (CTG), and these were compared to patients who did not receive chemotherapy (Time by group interaction) (n = 28). Only cell populations with statistically significant relationships are shown. *Naïve* CD45RA + CCR7 + CD27 + CD28+, *Central memory* CD45RA-CCR7 + CD27 + CD28+, *TEMRA* CD45RA + CCR7-CD27-CD28–. *P* values in bold are statistically significant

**Fig. 1:**
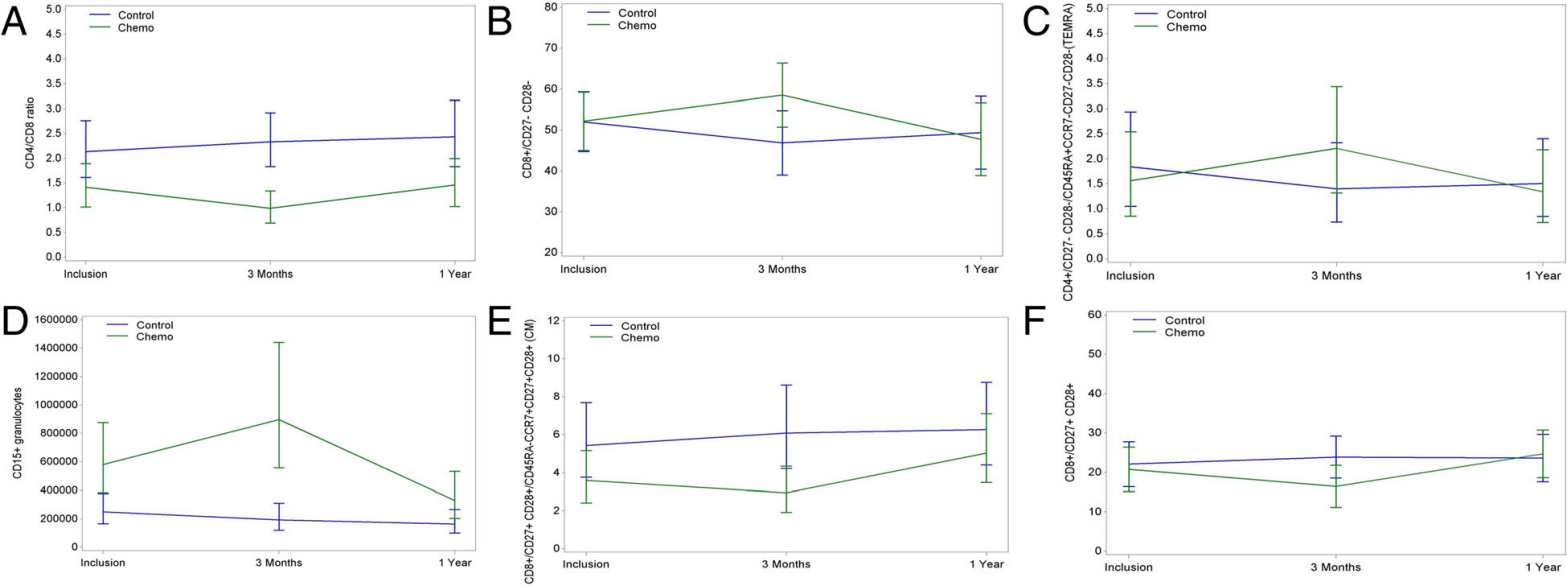
Estimated mean immune biomarker levels (with 95% confidence intervals) in patients undergoing chemotherapy (*green lines*) or not having chemotherapy (*blue lines*) at baseline, 3 months and 12 months after starting therapy. Relative to patients not receiving chemotherapy (control group (CG)), those receiving chemotherapy (chemotherapy group (CTG)) had a lower ratio of CD4:CD8 T cells at 3 months before partial normalization (**a**), while there were relative increases in CTG patients at 3 months followed by normalization at 12 months in CD8 + CD27-CD28– (**b**) CD4+ T effector memory re-expressing CD45RA **(**TEMRA) memory cells (**c**) and CD15+ granulocytic cells (**d**). There were relative decreases in CD8+ central memory cells (**e**) and CD8+ CD27+ CD28+ T cells (**f**) in the CTG at 3 months before normalization. *Chemo* chemotherapy group

We tested whether the evolution over time was different between the groups (Table 1, “Time by group interaction” shows the comparison between the CTG and CG groups), and identified chemotherapy-specific effects on the immune system. There were significant differences between the CTG and the CG groups in the ratio of CD4:CD8 T cells, which was slightly lower at 3 months in CTG patients before approaching normalization again after 1 year (Fig. 1a). This is in agreement with the observed increase of CD8+ cells at 3 months in the CTG but not the CG, and indicates a difference in the balance between CD4+ and CD8+ T cells. CD4+ cells are mostly “helper” cells but also include populations of Treg suppressor cells, while CD8+ T cells are mostly cytotoxic cells.

The evolution of CD8 + CD27-CD28– cells, CD4+ TEMRA memory cells and CD15+ granulocytic cells over time was also different in CTG and CT patients; it had increased by 3 months in the CTG, but not in the CG, before normalizing again at 1 year (Fig. 1b-d). There were changes in CD8+ central memory cells and CD8+ CD27+ CD28+ double-positive T cells in the opposite direction (i.e. relatively lower levels at 3 months before normalization at 1 year) in CTG patients compared with CG patients (Fig. 1e, f). A number of other cell populations also differed over time in CTG patients compared to CG patients (Table 1 provides an overview, and the full set of graphical results are in Additional file 6). Background information on these leukocyte populations is in Additional file 5.

### CMV status, plasma cytokines, and telomere length are associated with immune profiles in elderly breast patients with cancer

We tested for associations between patient immune profiles and markers of patient health status including leukocyte telomere length, CMV serostatus, circulating cytokines and chemokines and the growth factor IGF-1 in the entire patient cohort at baseline (i.e. the CTG and CG combined, prior to any systemic therapy). A number of leukocyte populations were related to plasma IL-6, IL-10, IGF-1, TNFα, MCP-1 and RANTES (Table 2). High IL-6 in blood has been linked to poor health status in numerous studies, so it was interesting that we observed inverse associations between plasma IL-6 and one MDSC population and putatively senescent CD4 + CD57+ T cells. There was also correlation between T cells and a number of other parameters. For instance, frequencies of CD8+ effector memory cells were inversely associated with telomere length, while there was positive correlation with CD4+ expressing Helios, a Treg population that remains poorly defined [12]. As expected based on numerous studies, CMV seropositivity was associated with lower frequencies of naïve and central memory T cells and higher frequencies of late-stage differentiated TEMRA and effector memory CD4+ T cells. The same was true in the CD8 compartment except in the case of effector memory cells, for which there was a trend towards higher frequency in CMV+ patients. Table 2 provides a comprehensive list of immune profiles that were significantly correlated with biomarkers of ageing.

**Table 2:** Associations between patient immune profile and biomarkers of ageing in the whole population (the CTG and CG) prior to receiving any systemic therapy

Feature	Phenotype	*p* Value	*r* Value	Number
Telomere length	CD8 + effector memory	0.003	*r* = –0.482	n = 36
Telomere length	CD4 + Helios+	0.031	*r* = 0.361	n = 36
IL-6	CD4 + CD57+	0.025	*r* = –0.316	n = 50
IL-6	CD3 + CD4+	0.034	*r* = 0.298	n = 51
IL-6	CD8 + Helios+	0.014	*r* = 0.343	n = 51
IL-6	Lin-CD14-HLA-DR-	0.014	*r* = –0.332	n = 54
IL-10	CD8+/TEMRA	0.030	*r* = 0.307	n = 50
IGF-1	CD4 + CD57+	0.035	*r* = 0.299	n = 50
IGF-1	CD4 + CD25hi FOXP3+	0.044	*r* = 0.283	n = 51
TNF	CD4 + CD25hi FOXP3+	0.019	*r* = –0.326	n = 51
TNF	CD4 + FOXP3+	0.045	*r* = –0.282	n = 51
TNF	CD8 + Helios+	0.025	*r* = 0.315	n = 51
TNF	CD14 + CD124+	0.003	*r* = 0.392	n = 54
TNF	CD15+	0.020	*r* = −0.317	n = 54
TNF	Lin-	0.009	*r* = 0.353	n = 54
TNF	Lin-CD14-	0.029	*r* = 0.297	n = 54
MCP-1	Lin-CD14+	0.018	*r* = 0.320	n = 54
RANTES	CD4+	0.017	*r* = –0.337	n = 50
RANTES	CD8+/TEMRA	0.012	*r* = −0.352	n = 50
RANTES	CD3 + CD4+	0.012	*r* = −0.350	n = 51
RANTES	CD8 + FOXP3++	0.001	*r* = 0.434	n = 51
RANTES	CD14-CD15 + CD124+	0.006	*r* = 0.370	n = 54
RANTES	Lin-CD14-HLA-DR-	0.009	*r* = 0.354	n = 54
CMV	CD4 + CD27+	0.031↓		n = 47
CMV	CD4 + CD27+ CD28+	0.044↓		n = 47
CMV	CD4+/Central memory	0.037↓		n = 47
CMV	CD4 + CD27- CD28-	<0.001↑		n = 47
CMV	CD4+/TEMRA	0.013↑		n = 47
CMV	CD4+/Effector memory	<0.001↑		n = 47
CMV	CD4 + CD28+	<0.001↑		n = 47
CMV	CD8+	0.020↑		n = 47
CMV	CD8 + CD27	0.021↓		n = 47
CMV	CD8 + CD27 + CD28+	0.008↓		n = 47
CMV	CD8+/Central memory	0.033↓		n = 47
CMV	CD8 + CD27- CD28-	<0.001↑		n = 47
CMV	CD8+/TEMRA	<0.001↑		n = 47
CMV	CD8 + CD28+	0.007↓		n = 47
CMV	CD4CD8 ratio	0.004↓		n = 47
CMV	CD8 + FOXP3++	0.039↓		n = 48

Negative *r* values indicate inverse association and positive *r* values indicate positive correlation between the patient feature (“Feature”) and the leukocyte population (“Phenotype”); ↓ indicates that cytomegalovirus (CMV) positive status is associated with lower values of the particular cell population and ↑ indicates higher values. ***CTG*** chemotherapy group, ***CG*** control group, ***IGF*** insulin-like growth factor, ***MCP*** monocyte chemattractant protein, ***RANTES*** regulated on activation, normal T cell expressed and secreted

### Patient immune profile is correlated with clinical frailty as measured by geriatric evaluation

In view of the correlation between immune profiles and health status biomarkers measured prior to therapy in these older patients with breast cancer, we also investigated whether the pre-treatment immune profile was influenced by age-related parameters (chronological age or clinical frailty) in the entire patient cohort. Within this elderly cohort, there was no correlation between any of the immune cell populations and chronological age by itself. On the contrary, when analyzing clinical frailty, patients with an inferior health status according to the LOFS had higher frequencies of granulocytic cells (these cells assist in the innate immune defense against pathogens and play a role in inflammation), but lower levels of granulocytic MDSCs and Tregs, both of which can suppress the immune system. Along these lines, patients with lower G8 scores also had lower levels of granulocytic MDSCs and Tregs (Table 3). Aside from these associations, we did not find any further relationships between clinical ageing and patients’ immune profiles.

**Table 3:** Patient immune profiles correlating with clinical frailty in the whole population (the CTG and CG) prior to receiving systemic therapy at baseline

Geriatric measure	Phenotype	*p* Value, *r* value, number
LOFS	CD4+/CD57+	0.0178, *r* = 0.341, n = 48
LOFS	CD8+/CD27	0.0367, *r* = 0.302, n = 48
LOFS	CD4+/CD25hi FOXP3+	0.0407, *r* = 0.291, n = 50
LOFS	CD4+/FOXP3+	0.0002, *r* = 0.510, n = 50
LOFS	CD14-negative/CD15+/CD124+	0.0292, *r* = 0.303, n = 52
LOFS	CD14-negative/HLA-DR-negative/CD11b+	0.0195, *r* = 0.323, n = 52
LOFS	CD15+	0.0246, *r* = -0.311, n = 52
G8	CD4+/FOXP3+	0.0147, *r* = 0.340, n = 51
G8	CD14-ve/HLA-DR-negative/CD11b+	0.0403, *r* = 0.280, n = 54

Negative *r* values indicate inverse association and positive *r* values show positive correlation between patient geriatric status (“Geriatric measure”) and the indicated leukocyte population (“Phenotype”). Lower G8 and Leuven oncology frailty score (LOFS) values designate more frail patients and high values indicate fitter patients. *CTG* chemotherapy group, *CG* control group

### Predictive value of immune profiles in patients receiving chemotherapy

Because we observed that chemotherapy induced changes in patient immune profiles and that clinical and biological health biomarkers were also related to immune profiles, we investigated whether patient immune profiles measured prior to chemotherapy administration predict decline in functionality, decline in quality of life, chemotherapy-related toxicity or unexpected hospitalizations during the 3-month treatment period. These analyses revealed that patients experiencing unexpected hospitalizations had lower levels of putatively suppressive granulocytic MDSCs (CD14–/CD15+/CD124+) (*p* = 0.037) but higher levels of other granulocytic cells (CD15+) (*p* = 0.049) (n = 26 for both). Aside from this, the immune profiles of chemotherapy patients were not associated with decline in functionality, decline in quality of life or chemotherapy-related toxicity.

## Discussion

The study of biomarkers in the elderly [9] was designed with the goal of identifying biomarkers that can accurately assess the health status of older patients with breast cancer and of investigating the impact of chemotherapy on these markers of biological and clinical ageing. For the first time, the present study combines not only geriatric assessment (GA), but also measures of blood-based biomarkers including leukocyte telomere length, plasma cytokines and growth factors [9], circulating microRNAs (manuscript submitted) and immune parameters such as CMV serostatus and circulating immune cell populations. A large-scale analysis of these multiple factors was performed in patients with breast cancer receiving chemotherapy and compared to patients receiving hormone treatment only.

The present report focuses on the immune biomarkers; it describes correlation between baseline cellular immune profiles and potential health indicators such as telomere length, CMV status and plasma cytokines and with the patients’ clinical frailty status as measured by GA. Immune profiles of patients receiving chemotherapy were additionally tested for their ability to predict decline in functionality, decline in quality of life, chemotherapy-related toxicity or unexpected hospitalizations. It was hypothesized that chemotherapy may have a significant short-term and/or longer-term effect on the immune system. Therefore, we assessed immune profiles before treatment and at 3 months and 12 months after the start of therapy, and compared the time-dependent evolution of diverse immune cell populations in patients treated with chemotherapy compared to patients who did not receive chemotherapy.

The potential limitations of this study must be acknowledged in that this study was exploratory, no power calculations were performed for the analyses reported and a large number of statistical tests were performed without correction for multiple testing. Therefore, these results require validation in an independent cohort. Nonetheless, the results of this study do support the hypothesis that there are therapy-dependent effects on the immune system.

Patients in the control group (CG) underwent surgery before inclusion in the study, which after inclusion was followed by anti-hormone therapy, and radiotherapy in most cases. In the chemotherapy group (CTG), the majority of patients also received radiotherapy and anti-hormone therapy, but all patients additionally received four cycles of docetaxel-cyclophosphamide. At 3 months and at 1 year after the start of systemic therapy, there were changes in the immune profiles of patients receiving chemotherapy. We observed elevated levels of total CD8+ T cells at 3 months and increased levels of naïve CD8+ T cells at 12 months after the start of chemotherapy, suggesting immune stimulation in chemotherapy-treated patients both immediately after the completion of chemotherapy and also in the longer term after the cessation of chemotherapy.

CD8+ T cells are primarily cytotoxic cells that provide immunological protection against viral and other infections, thereby revealing a potential mechanism of chemotherapy-induced immunomodulation. Naïve CD8+ T cells represent a primary pathway by which the adaptive immune system provides immunological protection against novel antigens. An increase in these cells may result in an enhanced capacity of these patients treated with chemotherapy to respond to novel tumor antigens; for example, during additional rounds of chemotherapy in patients who relapse, and together with chemotherapy-induced immunogenic cell death, may represent an additional immunological therapeutic mechanism of chemotherapy [13]. However, these apparent immune stimulatory effects may be balanced out by elevations in populations of suppressive cells; two populations of MDSCs were also increased after 3 months and 12 months in patients receiving chemotherapy. This suggests that in this clinical setting, MDSC-targeted therapies may result in improved patient status. This observation is also in contrast to the emerging view that at least some types of chemotherapy in certain cancers may act to selectively depress MDSC function and/or viability, as does doxorubicin in breast cancer [14], and hence contribute positively to the beneficial effects of such treatment [15]. In all likelihood, the end effect of these complex interactions will be highly context-dependent [16]. For example, hormone therapy has the potential to influence aspects of the immune system [17], which may relate to our observations here of therapy-dependent effects on the immune system; patients in the CG received hormone therapy as the sole form of systemic treatment, whereas the majority of patients in the CTG group received hormone therapy after chemotherapy.

The present study identified a large number of correlations between patient immune profile and biomarkers of patient biological age and health status, including CMV serostatus, leukocyte telomere length and circulating cytokines or growth factors. In accordance with numerous studies (reviewed in [18]), we observed lower frequencies of naïve and central memory T cells and higher frequencies of late-stage differentiated TEMRA and effector memory T cells in CMV-positive patients, in line with the notion that CMV infection contributes to immune exhaustion, particularly in elderly individuals. We also observed inverse correlation between IL-6 and the CD14-HLA-DR– MDSC population, but there was no correlation between IL-6 and other MDSC phenotypes. This suggests that the purported role of IL-6 in expanding suppressive myeloid cells [19] is not reflected in human samples analyzed directly ex vivo. Samples of this type are more likely to be indicative of human biology, as compared with prior studies, which have investigated the role of IL-6 in expanding MDSCs using animal models or in vitro cultures of samples of human peripheral blood mononuclear cells (PBMCs). IL-6 has been reported to be associated with poor health status in a number of previous studies (reviewed in [20]). The concomitant alterations in IL-6 and blood leukocytes suggest pathways through which IL-6 may be related to inferior health status.

The clinical age of these elderly patients with breast cancer, as measured with the GA scores LOFS and G8, was related to certain leukocyte populations in blood. The observation that patients with an inferior health status according to the LOFS had higher frequencies of granulocytic cells but lower levels of putatively suppressive granulocytic MDSCs and Tregs may identify patients who have an ongoing infection or who have high levels of inflammation mediated by granulocytic cells that is not dampened by MDSCs or Tregs, as would usually occur. Moreover, the combination of high levels of granulocytic cells and low levels of MDSCs measured at baseline was also related to the prediction of health issues in patients receiving chemotherapy. The finding that patients with unexpected hospitalizations had lower levels of putatively suppressive granulocytic MDSCs but higher levels of other granulocytic cells is intriguing, and may reflect the result of a latent infection or may be related to high levels of inflammation that have not been suppressed by this MDSC population.

## Conclusion

To the best of our knowledge, this is the first study to investigate the effect of chemotherapy on the immune system in elderly patients with breast cancer using ex vivo analyses, and also the first to assess the association between clinical frailty, biomarkers of ageing and immune profiles in this context. We report that elderly breast patients with cancer undergoing chemotherapy have alterations in their immune profiles over time. Furthermore, there is correlation between specific circulating leukocyte populations measured prior to therapy and biomarkers of ageing, including telomere length and blood cytokines, and clinical frailty scored by the LOFS and G8. The immune biomarkers identified in this study may therefore provide a novel tool for the assessment of health status in elderly patients with breast cancer, which may be further developed in the future to better guide the therapeutic management of patients with breast cancer on an individual basis.

## Additional files

Additional file 1: Clinical characteristics of the control and chemotherapy patient groups (DOCX 23 kb)
Additional file 2: Gating strategy used to identify T cell subsets and differentiation stages (PPTX 227 kb)
Additional file 3: Gating strategy used to identify regulatory T cells (PPTX 182 kb)
Additional file 4: Gating strategy used to identify myeloid cells including myeloid-derived suppressor cells (PPTX 189 kb)
Additional file 5: Leukocyte phenotypes examined in peripheral blood (PPTX 78 kb)
Additional file 6: Estimated mean blood leukocyte levels (with 95% confidence intervals) in patients undergoing chemotherapy (*green lines*) or without chemotherapy (*blue lines*) at baseline and at 3 months and 12 months after starting therapy (PPTX 1131 kb)

## Abbreviations

ADL: Katz’s activities of daily living
CCI: Charlson comorbidity index
CG: Control group
CMV: Cytomegalovirus
CTG: Chemotherapy group
ECOG-PS: Eastern Cooperative Oncology Group - performance status
EORTC QLQ-C30: European Organization for Research and Treatment for Cancer quality of life questionnaire
FBS: Fetal bovine serum
GA: Geriatric assessment
GDS: Geriatric depression scale
HER2: Human epidermal growth factor receptor 2
IADL: Lawton’s instrumental activities of daily living
IGF-1: Insulin-like growth factor 1
IL: Interleukin
Lin: Lineage
LOFS: Leuven oncology frailty score
MCP: Monocyte chemoattractant protein
MDSC: Myeloid-derived suppressor cell
MMSE: Mini mental state examination
MNA-SF: Mini nutritional assessment - short form
MOB-T: Mobility–tiredness test
PFEA: Phosphate-buffered saline, 2% FBS, 2 mM EDTA, 0.01% azide
QoL: Quality of life
RANTES: Regulated on activation, normal T cell expressed and secreted
TC: Docetaxel-cyclophosphamide
TEMRA: T effector memory re-expressing CD45RA
TL: Telomere length
TNF: Tumor necrosis factor
TNM: Tumor node metastasis
Tregs: Regulatory T cells
VAS: Visual analog scale
