# Supplementary material for: Immune profiles of elderly breast cancer patients are altered by chemotherapy and relate to clinical frailty
Source: Breast Cancer Res. 2017 Feb 28;19:20. doi: 10.1186/s13058-017-0813-x (PMC5330012; doi:10.1186/s13058-017-0813-x)
Supplement: Additional file 1: — Clinical characteristics of the control and chemotherapy patient groups (DOCX 23 kb) [file 13058_2017_813_MOESM1_ESM.docx]

|  | **Control group** | **Chemotherapy group** |
| --- | --- | --- |
| **Age** | **n=28** | **n=28** |
| Median, years (range) | 75.0 (70.0-88.0) | 73.0 (70.0-80.0) |
| **pT** | **n=28** | **n=27** |
| 1 | 11/28 (39.3%) | 6/27 (22.2%) |
| 2 | 17/28 (60.7%) | 18/27 (66.7%) |
| 3 | 0/28 (0.0%) | 3/27 (11.1%) |
| **pN** | **n=27** | **n=26** |
| 0 | 15/27 (55.6%) | 9/26 (34.6%) |
| 1-3 | 12/27 (44.4%) | 17/26 (65.4%) |
| **Grade** | **n=25** | **n=27** |
| 2 | 17/25 (68.0%) | 6/27 (22.2%) |
| 3 | 8/25 (32.0%) | 21/27 (77.8%) |
| **Estrogen receptor (ER)** | **n=28** | **n=27** |
| Positive | 28/28 (100%) | 18/27 (66.7%) |
| Negative | 0/28 (0.0%) | 9/27 (33.3%) |
| **Progesteron receptor (PR)** | **n=28** | **n=27** |
| Positive | 24/28 (85.7%) | 13/27 (48.2%) |
| Negative | 4/28 (14.3%) | 14/27 (51.8%) |
| **HER2 receptor** | **n=28** | **n=26** |
| Positive | 1/28 (3.6%) | 6/26 (23.1%) |
| Negative | 27/28 (96.4%) | 20/26 (76.2%) |
| **Breast cancer phenotype*** | **n=28** | **n=27** |
| Basal-like | 0/28 (0.0%) | 8/27 (29.6%) |
| HER2 positive | 0/28 (0.0%) | 2/27 (7.4%) |
| Luminal A | 20/28 (71.4%) | 4/27 (14.8%) |
| Luminal B HER2 negative | 7/28 (25.0%) | 9/27 (33.3%) |
| Luminal B HER2 positive | 1/28 (3.6%) | 4/27 (14.8%) |
| **Adjuvant therapy** | **n=28** | **n=27** |
| TC chemotherapy | 0/28 (0.0%) | 27/27 (100%) |
| G-CSF primary prophylaxis | 0/28 (0.0%) | 26/27 (96.3%) |
| Trastuzumab 1 year | 0/28 (0.0%) | 6/27 (22.2%) |
| Endocrine therapy | 28/28 (100%) | 17/27 (63.0%) |
| Radiotherapy | 22/28 (78.6%) | 23/27 (85.2%) |
| **LOFS** | **n=27** | **n=27** |
| Range (0=very frail, 10=very fit) | 2.0-10.0 | 5.0-10.0 |
| Mean ± SD | 7.2 ± 2.4 | 7.7 ± 1.3 |
| **G8** | **n=24** | **n=26** |
| Range (0=very frail, 17=very fit) | 10.0-17.0 | 9.0-17.0 |
| Mean ± SD | 14.0 ± 1.9 | 14.5 ± 1.9 |
| **Unexpected hospitalizations** | **n=28** | **n=28** |
| No (3 months) | 26/28 (92.9%) | 19/28 (67.9%) |
| Yes (3 months) | 2/28 (7.1%) | 8/28 (28.6%) |
| Unknown (3 months) | 0/28 (0.0%) | 1/28 (3.6%) |
| No (12 months) | 21/28 (75.0%) | 22/28 (78.6%) |
| Yes (12 months) | 7/28 (25.0%) | 5/28 (17.9%) |
| Unknown (12 months) | 0/28 (0.0%) | 1/28 (3.6%) |

Additional File 1: Clinical characteristics of the control and chemotherapy patient groups.

Abbreviations: TC, Docetaxel-Cyclophosphamide; G-CSF, Granulocyte-Colony Stimulating Factor; LOFS, Leuven Oncology Frailty Score

* Breast cancer phenotype definition: for details see Goldhirsch, A. *et al*. Ann Oncol. 2011;22(8):1736-1747.
