# Supplementary material for: Immune profiles of elderly breast cancer patients are altered by chemotherapy and relate to clinical frailty
Source: Breast Cancer Res. 2017 Feb 28;19:20. doi: 10.1186/s13058-017-0813-x (PMC5330012; doi:10.1186/s13058-017-0813-x)
Supplement: Additional file 2: — Gating strategy used to identify T cell subsets and differentiation stages (PPTX 227 kb) [file 13058_2017_813_MOESM2_ESM.pptx]

## Slide 1
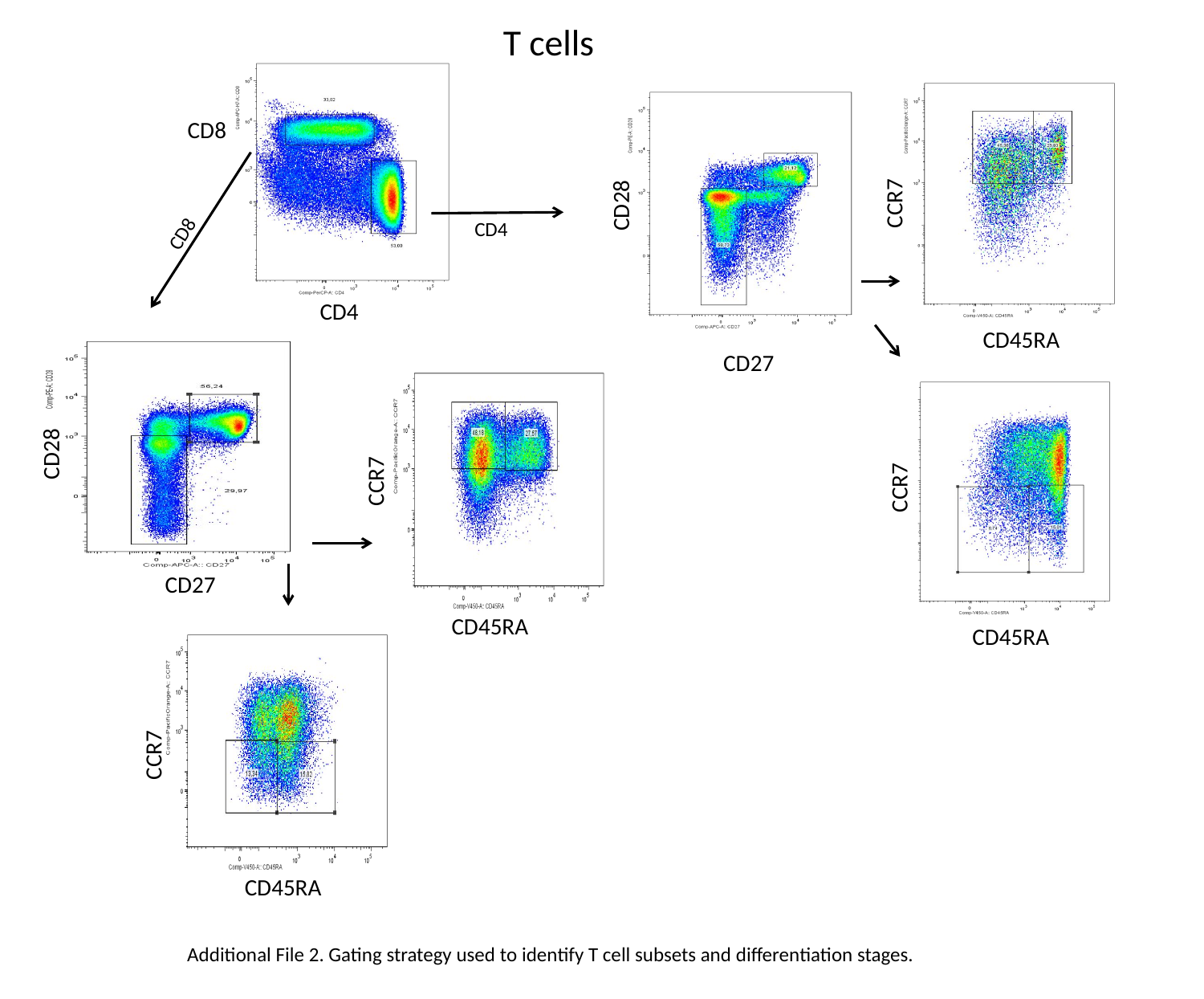

T cells
CD8
CD4
CCR7
CD45RA
CD28
CD27
CD28
 CD27
CCR7
CD45RA
 CCR7
CD45RA
CCR7
CD45RA
CD4
CD8
Additional File 2. Gating strategy used to identify T cell subsets and differentiation stages.
