# Supplementary material for: Immune profiles of elderly breast cancer patients are altered by chemotherapy and relate to clinical frailty
Source: Breast Cancer Res. 2017 Feb 28;19:20. doi: 10.1186/s13058-017-0813-x (PMC5330012; doi:10.1186/s13058-017-0813-x)
Supplement: Additional file 3: — Gating strategy used to identify regulatory T cells (PPTX 182 kb) [file 13058_2017_813_MOESM3_ESM.pptx]

## Slide 1
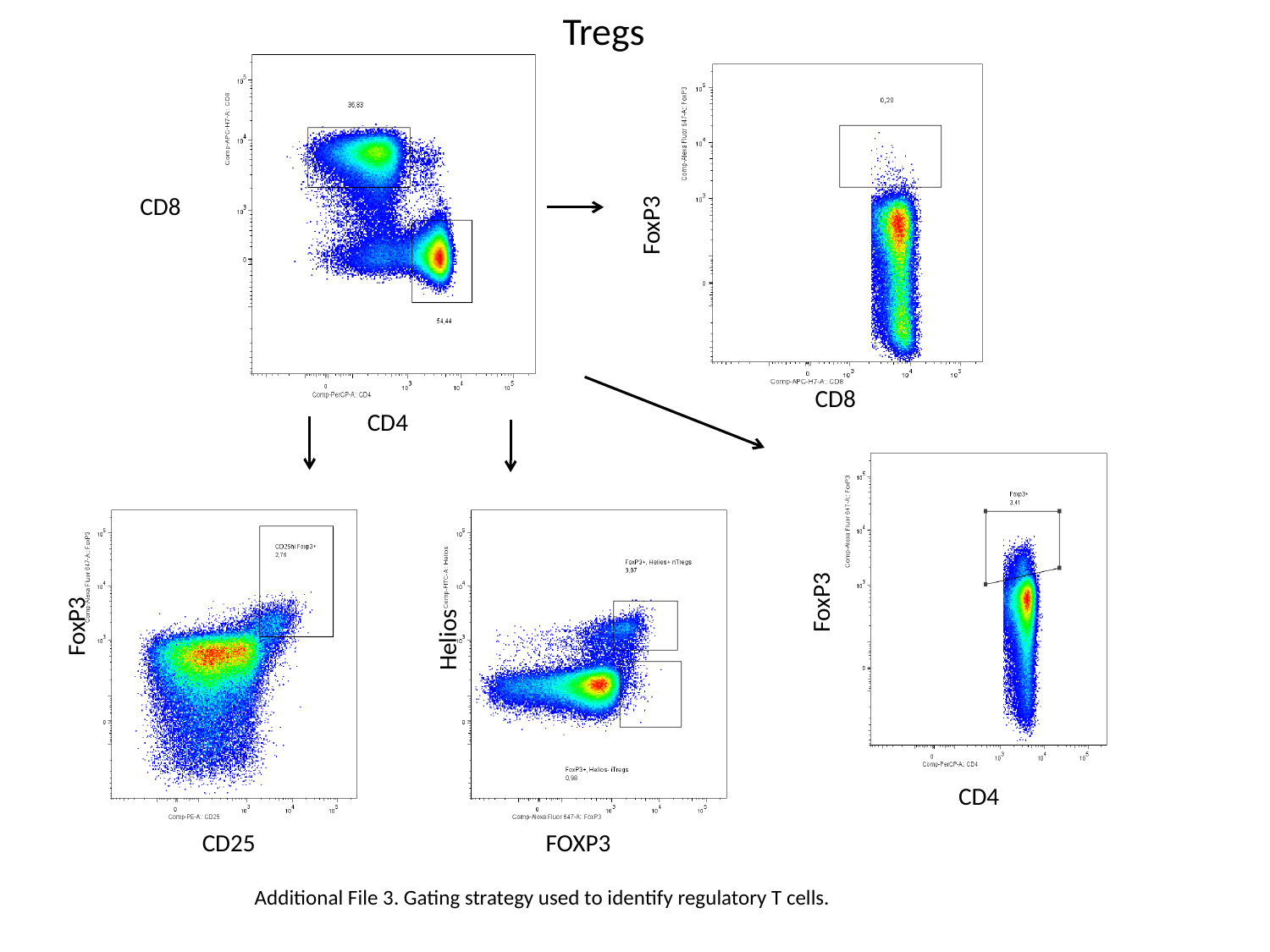

Tregs
CD8
CD4
 FoxP3
CD8
 FoxP3
CD4
FoxP3
CD25
Helios
FOXP3
Additional File 3. Gating strategy used to identify regulatory T cells.
