# Supplementary material for: Immune profiles of elderly breast cancer patients are altered by chemotherapy and relate to clinical frailty
Source: Breast Cancer Res. 2017 Feb 28;19:20. doi: 10.1186/s13058-017-0813-x (PMC5330012; doi:10.1186/s13058-017-0813-x)
Supplement: Additional file 4: — Gating strategy used to identify myeloid cells including myeloid-derived suppressor cells (PPTX 189 kb) [file 13058_2017_813_MOESM4_ESM.pptx]

## Slide 1
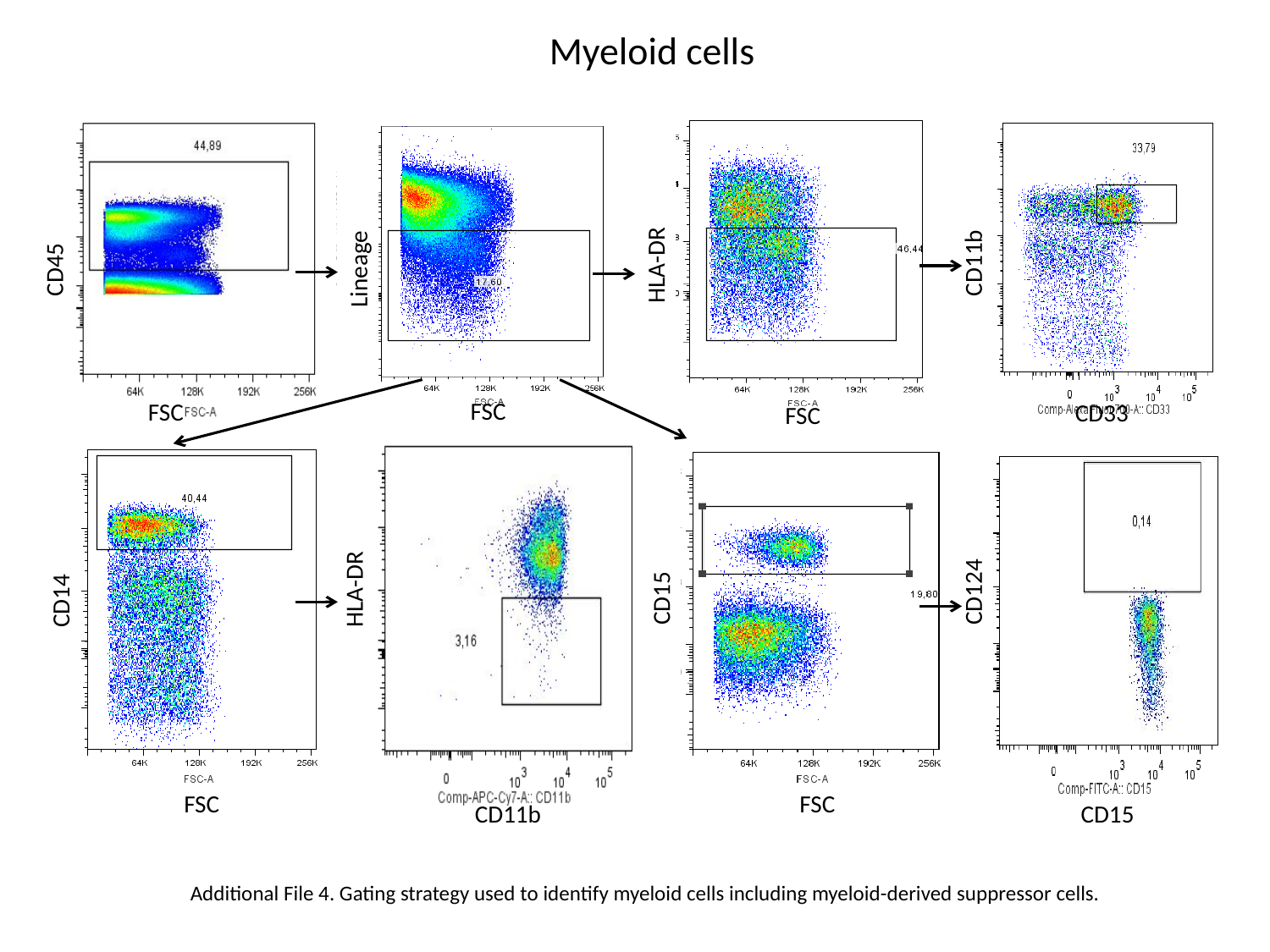

Myeloid cells
 CD11b
 Lineage
 HLA-DR
 CD45
FSC
FSC
CD33
FSC
 CD14
 HLA-DR
 CD15
 CD124
FSC
FSC
CD15
CD11b
Additional File 4. Gating strategy used to identify myeloid cells including myeloid-derived suppressor cells.
