# Supplementary material for: Immune profiles of elderly breast cancer patients are altered by chemotherapy and relate to clinical frailty
Source: Breast Cancer Res. 2017 Feb 28;19:20. doi: 10.1186/s13058-017-0813-x (PMC5330012; doi:10.1186/s13058-017-0813-x)
Supplement: Additional file 5: — Leukocyte phenotypes examined in peripheral blood (PPTX 78 kb) [file 13058_2017_813_MOESM5_ESM.pptx]

## Slide 1
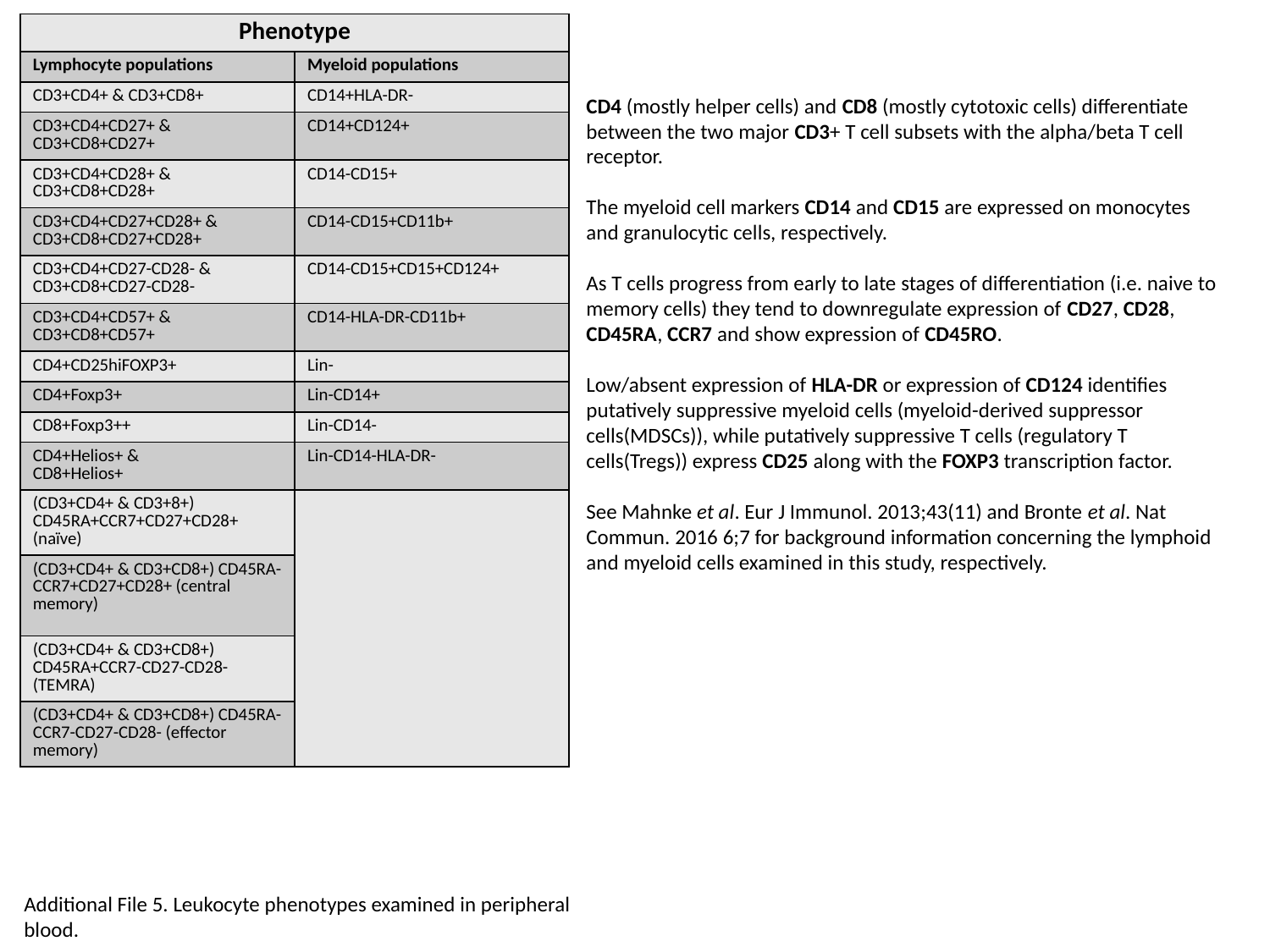

| Phenotype | |
| --- | --- |
| Lymphocyte populations | Myeloid populations |
| CD3+CD4+ & CD3+CD8+ | CD14+HLA-DR- |
| CD3+CD4+CD27+ & CD3+CD8+CD27+ | CD14+CD124+ |
| CD3+CD4+CD28+ & CD3+CD8+CD28+ | CD14-CD15+ |
| CD3+CD4+CD27+CD28+ & CD3+CD8+CD27+CD28+ | CD14-CD15+CD11b+ |
| CD3+CD4+CD27-CD28- & CD3+CD8+CD27-CD28- | CD14-CD15+CD15+CD124+ |
| CD3+CD4+CD57+ & CD3+CD8+CD57+ | CD14-HLA-DR-CD11b+ |
| CD4+CD25hiFOXP3+ | Lin- |
| CD4+Foxp3+ | Lin-CD14+ |
| CD8+Foxp3++ | Lin-CD14- |
| CD4+Helios+ & CD8+Helios+ | Lin-CD14-HLA-DR- |
| (CD3+CD4+ & CD3+8+) CD45RA+CCR7+CD27+CD28+ (naïve) | |
| (CD3+CD4+ & CD3+CD8+) CD45RA-CCR7+CD27+CD28+ (central memory) | |
| (CD3+CD4+ & CD3+CD8+) CD45RA+CCR7-CD27-CD28- (TEMRA) | |
| (CD3+CD4+ & CD3+CD8+) CD45RA-CCR7-CD27-CD28- (effector memory) | |
CD4 (mostly helper cells) and CD8 (mostly cytotoxic cells) differentiate between the two major CD3+ T cell subsets with the alpha/beta T cell receptor.
The myeloid cell markers CD14 and CD15 are expressed on monocytes and granulocytic cells, respectively.
As T cells progress from early to late stages of differentiation (i.e. naive to memory cells) they tend to downregulate expression of CD27, CD28, CD45RA, CCR7 and show expression of CD45RO.
Low/absent expression of HLA-DR or expression of CD124 identifies putatively suppressive myeloid cells (myeloid-derived suppressor cells(MDSCs)), while putatively suppressive T cells (regulatory T cells(Tregs)) express CD25 along with the FOXP3 transcription factor.
See Mahnke et al. Eur J Immunol. 2013;43(11) and Bronte et al. Nat Commun. 2016 6;7 for background information concerning the lymphoid and myeloid cells examined in this study, respectively.
Additional File 5. Leukocyte phenotypes examined in peripheral blood.
