# Supplementary material for: Immune profiles of elderly breast cancer patients are altered by chemotherapy and relate to clinical frailty
Source: Breast Cancer Res. 2017 Feb 28;19:20. doi: 10.1186/s13058-017-0813-x (PMC5330012; doi:10.1186/s13058-017-0813-x)
Supplement: Additional file 6: — Estimated mean blood leukocyte levels (with 95% confidence intervals) in patients undergoing chemotherapy (green lines) or without chemotherapy (blue lines) at baseline and at 3 months and 12 months after starting therapy (PPTX 1131 kb) [file 13058_2017_813_MOESM6_ESM.pptx]

## Slide 1
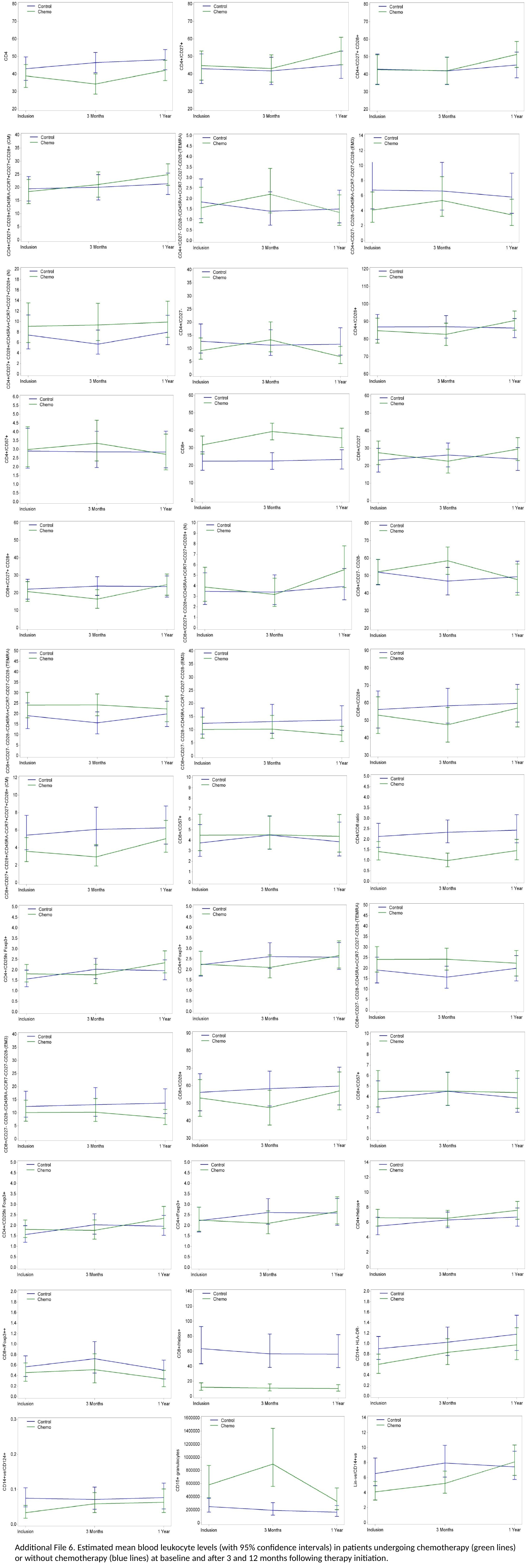

Additional File 6. Estimated mean blood leukocyte levels (with 95% confidence intervals) in patients undergoing chemotherapy (green lines) or without chemotherapy (blue lines) at baseline and after 3 and 12 months following therapy initiation.
